# 6-Cyanona­phthalen-2-yl 4-hexyl­benzo­ate

**DOI:** 10.1107/S160053681400909X

**Published:** 2014-04-30

**Authors:** Md. Lutfor Rahman, H. T. Srinivasa, Mohd. Yusoff Mashitah, Huey Chong Kwong, Ching Kheng Quah

**Affiliations:** a University Malaysia Pahang, Faculty of Industrial Sciences and Technology, 26300 Gambang, Kuantan, Pahang, Malaysia; b Raman Research Institute, C.V. Raman Avenue, Sadashivanagar, Bangalore 560080, India; c School of Chemistry Sciences, Universiti Sains Malaysia, 11800 USM, Penang, Malaysia; d X-ray Crystallography Unit, School of Physics, Universiti Sains Malaysia, 11800 USM, Penang, Malaysia

## Abstract

In the title compound, C_24_H_23_NO_2_, a whole mol­ecule is disordered over two sets of sites with occupancies in a ratio of 0.692 (6):0.308 (6). In the major disorder component, the naphthalene ring system forms a dihedral angle of 68.6 (5)° with the benzene ring. The corresponding angle in the minor component is 81.6 (10)°. In the crystal, mol­ecules are linked into chains propagating along the *b*-axis direction *via* weak C—H⋯O hydrogen bonds. The crystal packing is further consolidated by weak C—H⋯π inter­actions.

## Related literature   

For features of electro-optical display devices, see: Cox & Clecak (1976 ▶); Reddy & Tschierske (2006 ▶); Hanasaki *et al.* (2011 ▶) For applications of cyano groups in liquid crystal displays, see: Coates & Gray (1976 ▶); Klingbiel *et al.* (1974 ▶); Takezoe & Takanishi (2006 ▶). For related structures, see: Kuzmina *et al.* (2010 ▶); Blake *et al.* (1995 ▶); Li (2006 ▶). For standard bond-length data, see: Allen *et al.* (1987 ▶).
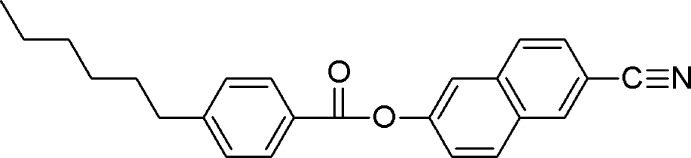


## Experimental   

### 

#### Crystal data  

C_24_H_23_NO_2_
*M* *_r_* = 357.43
Monoclinic, 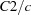

*a* = 14.4712 (2) Å
*b* = 9.5592 (2) Å
*c* = 29.5386 (5) Å
β = 98.898 (1)°
*V* = 4036.99 (12) Å^3^
*Z* = 8
Cu *K*α radiation
μ = 0.59 mm^−1^
*T* = 298 K
0.29 × 0.11 × 0.08 mm

#### Data collection  

Bruker SMART APEXII CCD area-detector diffractometer
Absorption correction: multi-scan (*SADABS*; Bruker, 2009 ▶) *T*_min_ = 0.848, *T*_max_ = 0.954
15700 measured reflections
3574 independent reflections
2344 reflections with *I* > 2σ(*I*)
*R*_int_ = 0.026

#### Refinement  

*R*[*F* ^2^ > 2σ(*F* ^2^)] = 0.069
*wR*(*F* ^2^) = 0.268
*S* = 1.10
3574 reflections
348 parameters
73 restraints
H-atom parameters constrained
Δρ_max_ = 0.25 e Å^−3^
Δρ_min_ = −0.22 e Å^−3^

### 

Data collection: *APEX2* (Bruker, 2009 ▶); cell refinement: *SAINT* (Bruker, 2009 ▶); data reduction: *SAINT*; program(s) used to solve structure: *SHELXTL* (Sheldrick, 2008 ▶); program(s) used to refine structure: *SHELXTL*; molecular graphics: *SHELXTL*; software used to prepare material for publication: *SHELXTL* and *PLATON* (Spek, 2009 ▶).

## Supplementary Material

Crystal structure: contains datablock(s) I. DOI: 10.1107/S160053681400909X/lh5699sup1.cif

Structure factors: contains datablock(s) I. DOI: 10.1107/S160053681400909X/lh5699Isup2.hkl

Supporting information file. DOI: 10.1107/S160053681400909X/lh5699Isup3.cml

CCDC reference: 998819

Additional supporting information:  crystallographic information; 3D view; checkCIF report

## Figures and Tables

**Table 1:** Hydrogen-bond geometry (Å, °) *Cg*1 and *Cg*2 are the centroids of the C2*A*–C5*A*/C10*A*/C11*A* and C13*B*–C18*B* rings, respectively.

*D*—H⋯*A*	*D*—H	H⋯*A*	*D*⋯*A*	*D*—H⋯*A*
C4*A*—H4*AA*⋯O2*A* ^i^	0.95	2.44	3.303 (11)	149
C9*B*—H9*BA*⋯O2*B* ^ii^	0.95	2.59	3.352 (11)	138
C14*B*—H14*B*⋯*Cg*1^iii^	0.95	2.85	3.708 (14)	151
C20*A*—H20*A*⋯*Cg*2^iv^	0.99	2.91	3.819 (14)	152
C19*B*—H19*C*⋯*Cg*2^iv^	0.99	2.88	3.746 (19)	146

Symmetry codes: (i) 
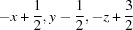
; (ii) 
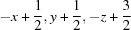
; (iii) 
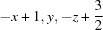
; (iv) 
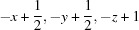
.

**Fig. 1.**
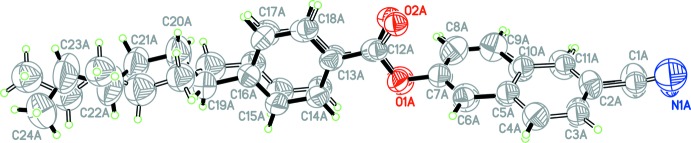
: The molecular structure of the title compound showing 30% probability displacement ellipsoids for the major component of disorder. Open bonds show the minor disordered component.

**Fig. 2.**
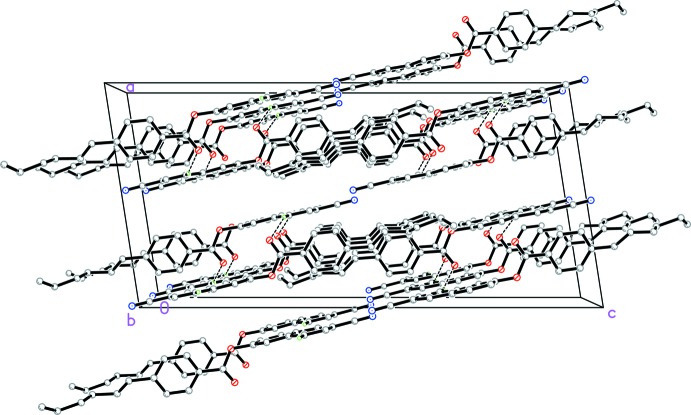
: The crystal packing of the title compound. Dashed lines represent the intermolecular hydrogen bonds. Only major disordered component is shown.

## supplementary crystallographic information

### 1. Comment

Electro-optical display devices require certain desirable features such as large positive dielectric anisotropy, nematic phase, low melting point, stability and lack of color (Cox & Clecak, 1976; Reddy & Tschierske, 2006; Hanasaki *et al.*, 2011). To obtain such properties, a highly polar terminal cyano group can be incorporated to give a large dipole moment. Maximum dipole moment (90 degrees) indicates that the dipole moment is exactly parallel to the molecular short axis, which acts along the long axis of the molecule and helps to give the proper alignment for liquid crystal displays (Coates & Gray, 1976; Klingbiel *et al.*, 1974; Takezoe & Takanishi, 2006). Here we report the synthesis and single-crystal X-ray study of an unsymmetrical naphthalene liquid crystal molecule. The shows a nematic phase after 379 K then a stable phase until an isotropic state at 411 K on a heating cycle. Upon cooling from the isotropic state, the nematic phase was reformed at 410 K, the phase is stabilized before crystallizes at 321 K.

The molecular structure of the title compound is shown in Fig 1. The whole molecule of the title compound is disordered over two positions with a refined site-occupancy ratio of 0.692 (6): 0.308 (6). For the major component, the naphthalene ring system (C2A—C11A) makes a dihedral angle of 68.6 (5)° with the benzene ring (C13A—C18A). In the minor component, the dihedral angle formed between the naphthalene ring system (C2B—C11B) and the benzene ring (C13B—C18B) is 81.6 (10)°. All the bond lengths (Allen *et al.*, 1987) and angles are in normal ranges and compared with the closely related structures (Kuzmina *et al.*, 2010; Blake *et al.*, 1995; Li, 2006)

In the crystal, molecules are linked into chains propagating along the *b*-axis *via* weak C—H···O hydrogen bonds. Weak C—H···π interactions are also observed (see Table 1).

### 2. Experimental

A mixture of 4-hexylbenzoic acid (1.0 mmol), 2-cyano-6-hydroxy-naphthalene (1.0 mmol), dicyclohexylcarbodiimide (1.2 mmol) and catalytic quantity of *N,N*-dimethylaminopyridine in 5 ml of dry dichloromethane was stirred at room temperature for 1 h. Progress of the reaction was monitored by TLC (ethyl acetate: pet ether 2:8). After completion of the reaction, the reaction mass was diluted with water and extracted into dichloromethane (25 ml). The organic layer was washed with diluted acetic acid and water. The organic layer was dried over anhydrous sodium sulfate and the solvent was removed under reduced pressure. The crude product was purified by column chromatography by using ethyl acetate: petroleum ether (2:8) as eluent and the product was recrystallization from chloroform. Yield = 70% as colourless block crystals. IR(KBr): *v* = 2920, 2856, 2224, 1724, 1454, 1066, 902 cm^-1^; ^1^H NMR (400 MHz, CDCl_3_): δ = 8.11–6.98 (m, 10H, Ar—H), 2.54 (t, *J* = 1.37 Hz, 2H, Ar—CH_2_–), 1.47–1.44 (m, 8H, alkyl-CH_2_–), 0.91 (m, 3H, alkyl-CH_3_) p.p.m.; Elemental analysis calcd for C_24_H_23_NO_2_ (%): C 80.64, H 6.49, N 3.92; found. C 80.69, H 6.54, N 4.07.

### 3. Refinement

The title compound is disordered over two positions with a refined site-occupancy ratio of 0.692 (6): 0.308 (6) and the minor disordered component was refined isotropically. All H atoms were positioned geometrically [C—H = 0.95–0.99 Å] and refined using a riding model with *U*_iso_(H) = 1.2 or 1.5 *U*_eq_(C). A rotating group model was applied to the methyl groups. The restraints of same geometries were applied to all disordered components. Identical anisotropic displacement and distance restraint were used in the final refinement. Similarity were applied to the disordered atoms. *DFIX* restraints of 1.50 (1) Å were used for the long-disordered alkyl chains such as C19B—C20B, C21B—C22B, C22B—C23B, C23B—C24B and C23A—C24A distances. Same *U^ij^* parameters restraints were used for C22A/C23A and C22B/C23B atom pairs. One outlier (1 1 10) was omitted from the reflection data.

### Crystal data

**Table:**

C_24_H_23_NO_2_	*F*(000) = 1520
*M**_r_* = 357.43	*D*_x_ = 1.176 Mg m^−^^3^
Monoclinic, *C*2/*c*	Cu *K*α radiation, λ = 1.54178 Å
Hall symbol: -C 2yc	Cell parameters from 5436 reflections
*a* = 14.4712 (2) Å	θ = 3.0–59.5°
*b* = 9.5592 (2) Å	µ = 0.59 mm^−^^1^
*c* = 29.5386 (5) Å	*T* = 298 K
β = 98.898 (1)°	Block, colourless
*V* = 4036.99 (12) Å^3^	0.29 × 0.11 × 0.08 mm
*Z* = 8	

### Data collection

**Table:**

Bruker SMART APEXII CCD area-detector diffractometer	3574 independent reflections
Radiation source: fine-focus sealed tube	2344 reflections with *I* > 2σ(*I*)
Graphite monochromator	*R*_int_ = 0.026
φ and ω scans	θ_max_ = 67.5°, θ_min_ = 3.0°
Absorption correction: multi-scan (*SADABS*; Bruker, 2009)	*h* = −17→17
*T*_min_ = 0.848, *T*_max_ = 0.954	*k* = −11→10
15700 measured reflections	*l* = −35→34

### Refinement

**Table:**

Refinement on *F*^2^	Primary atom site location: structure-invariant direct methods
Least-squares matrix: full	Secondary atom site location: difference Fourier map
*R*[*F*^2^ > 2σ(*F*^2^)] = 0.069	Hydrogen site location: inferred from neighbouring sites
*wR*(*F*^2^) = 0.268	H-atom parameters constrained
*S* = 1.10	*w* = 1/[σ^2^(*F*_o_^2^) + (0.1621*P*)^2^ + 0.4013*P*] where *P* = (*F*_o_^2^ + 2*F*_c_^2^)/3
3574 reflections	(Δ/σ)_max_ = 0.001
348 parameters	Δρ_max_ = 0.25 e Å^−^^3^
73 restraints	Δρ_min_ = −0.22 e Å^−^^3^

### Special details

**Table:**

Geometry. All e.s.d.'s (except the e.s.d. in the dihedral angle between two l.s. planes) are estimated using the full covariance matrix. The cell e.s.d.'s are taken into account individually in the estimation of e.s.d.'s in distances, angles and torsion angles; correlations between e.s.d.'s in cell parameters are only used when they are defined by crystal symmetry. An approximate (isotropic) treatment of cell e.s.d.'s is used for estimating e.s.d.'s involving l.s. planes.
Refinement. Refinement of *F*^2^ against ALL reflections. The weighted *R*-factor *wR* and goodness of fit *S* are based on *F*^2^, conventional *R*-factors *R* are based on *F*, with *F* set to zero for negative *F*^2^. The threshold expression of *F*^2^ > σ(*F*^2^) is used only for calculating *R*-factors(gt) *etc*. and is not relevant to the choice of reflections for refinement. *R*-factors based on *F*^2^ are statistically about twice as large as those based on *F*, and *R*- factors based on ALL data will be even larger.

### Fractional atomic coordinates and isotropic or equivalent isotropic displacement parameters (Å^2^)

**Table:**

	*x*	*y*	*z*	*U*_iso_*/*U*_eq_	Occ. (<1)
N1A	0.4778 (8)	0.1930 (11)	0.99962 (18)	0.145 (3)	0.692 (6)
C1A	0.4583 (9)	0.1754 (13)	0.9609 (2)	0.161 (4)	0.692 (6)
C2A	0.4351 (12)	0.1379 (10)	0.9130 (2)	0.121 (5)	0.692 (6)
C3A	0.4307 (11)	−0.0035 (9)	0.8995 (3)	0.125 (5)	0.692 (6)
H3AA	0.4415	−0.0758	0.9218	0.150*	0.692 (6)
C4A	0.4110 (9)	−0.0350 (9)	0.8543 (3)	0.130 (4)	0.692 (6)
H4AA	0.4045	−0.1304	0.8453	0.156*	0.692 (6)
C5A	0.3996 (15)	0.0695 (7)	0.8201 (2)	0.095 (5)	0.692 (6)
C6A	0.3754 (5)	0.0381 (5)	0.77329 (15)	0.090 (2)	0.692 (6)
H6AA	0.3707	−0.0566	0.7635	0.108*	0.692 (6)
C7A	0.3590 (10)	0.1417 (10)	0.7424 (2)	0.113 (5)	0.692 (6)
C8A	0.3659 (11)	0.2835 (11)	0.7551 (3)	0.154 (5)	0.692 (6)
H8AA	0.3557	0.3550	0.7325	0.185*	0.692 (6)
C9A	0.3874 (10)	0.3163 (8)	0.8002 (3)	0.146 (5)	0.692 (6)
H9AA	0.3895	0.4117	0.8093	0.175*	0.692 (6)
C10A	0.4066 (15)	0.2110 (8)	0.8340 (2)	0.109 (4)	0.692 (6)
C11A	0.4239 (8)	0.2407 (9)	0.8809 (3)	0.137 (4)	0.692 (6)
H11A	0.4280	0.3356	0.8906	0.164*	0.692 (6)
O1A	0.3432 (3)	0.1110 (9)	0.69588 (13)	0.116 (3)	0.692 (6)
O2A	0.1939 (3)	0.1789 (6)	0.69048 (16)	0.1168 (13)	0.692 (6)
C12A	0.2548 (5)	0.1298 (13)	0.6719 (2)	0.122 (4)	0.692 (6)
C13A	0.2471 (4)	0.0879 (13)	0.62349 (17)	0.099 (2)	0.692 (6)
C14A	0.3195 (3)	0.0298 (8)	0.60548 (14)	0.1111 (15)	0.692 (6)
H14A	0.3777	0.0133	0.6245	0.133*	0.692 (6)
C15A	0.3080 (3)	−0.0047 (8)	0.55975 (13)	0.1260 (17)	0.692 (6)
H15A	0.3590	−0.0449	0.5477	0.151*	0.692 (6)
C16A	0.2256 (3)	0.0168 (8)	0.53108 (13)	0.1217 (16)	0.692 (6)
C17A	0.1539 (5)	0.0786 (19)	0.5497 (2)	0.134 (4)	0.692 (6)
H17A	0.0962	0.0977	0.5306	0.161*	0.692 (6)
C18A	0.1643 (5)	0.1128 (14)	0.5950 (2)	0.137 (4)	0.692 (6)
H18A	0.1137	0.1544	0.6070	0.164*	0.692 (6)
C19A	0.2169 (5)	−0.0254 (9)	0.48135 (16)	0.171 (3)	0.692 (6)
H19A	0.2802	−0.0236	0.4726	0.206*	0.692 (6)
H19B	0.1948	−0.1235	0.4787	0.206*	0.692 (6)
C20A	0.1562 (6)	0.0569 (8)	0.44850 (17)	0.198 (3)	0.692 (6)
H20A	0.1738	0.1563	0.4536	0.237*	0.692 (6)
H20B	0.0916	0.0465	0.4553	0.237*	0.692 (6)
C21A	0.1545 (5)	0.0258 (10)	0.39913 (17)	0.181 (3)	0.692 (6)
H21A	0.1073	0.0879	0.3815	0.218*	0.692 (6)
H21B	0.2161	0.0524	0.3912	0.218*	0.692 (6)
C22A	0.1342 (9)	−0.1186 (11)	0.3829 (2)	0.250 (4)	0.692 (6)
H22A	0.0863	−0.1630	0.3987	0.300*	0.692 (6)
H22B	0.1914	−0.1770	0.3872	0.300*	0.692 (6)
C23A	0.0965 (9)	−0.0943 (9)	0.3306 (2)	0.250 (4)	0.692 (6)
H23A	0.0277	−0.0830	0.3250	0.300*	0.692 (6)
H23B	0.1261	−0.0117	0.3185	0.300*	0.692 (6)
C24A	0.1261 (7)	−0.2275 (10)	0.3103 (3)	0.231 (4)	0.692 (6)
H24A	0.1177	−0.2188	0.2768	0.347*	0.692 (6)
H24B	0.0879	−0.3050	0.3187	0.347*	0.692 (6)
H24C	0.1921	−0.2458	0.3220	0.347*	0.692 (6)
N1B	0.459 (2)	0.187 (4)	1.0011 (6)	0.194 (12)*	0.308 (6)
C1B	0.4572 (10)	0.1627 (15)	0.9629 (3)	0.087 (3)*	0.308 (6)
C2B	0.441 (2)	0.1329 (12)	0.9143 (3)	0.088 (6)*	0.308 (6)
C3B	0.4330 (17)	−0.0078 (13)	0.8999 (4)	0.090 (7)*	0.308 (6)
H3BA	0.4409	−0.0813	0.9218	0.108*	0.308 (6)
C4B	0.4144 (10)	−0.0374 (12)	0.8545 (3)	0.077 (4)*	0.308 (6)
H4BA	0.4156	−0.1316	0.8444	0.092*	0.308 (6)
C5B	0.393 (4)	0.0715 (14)	0.8222 (4)	0.095 (12)*	0.308 (6)
C6B	0.379 (2)	0.0423 (19)	0.7750 (4)	0.149 (11)*	0.308 (6)
H6BA	0.3777	−0.0518	0.7647	0.179*	0.308 (6)
C7B	0.366 (2)	0.1472 (15)	0.7444 (4)	0.099 (9)*	0.308 (6)
C8B	0.3689 (13)	0.2883 (13)	0.7582 (4)	0.094 (5)*	0.308 (6)
H8BA	0.3570	0.3608	0.7360	0.112*	0.308 (6)
C9B	0.3894 (12)	0.3195 (13)	0.8036 (4)	0.093 (5)*	0.308 (6)
H9BA	0.3970	0.4144	0.8129	0.112*	0.308 (6)
C10B	0.399 (3)	0.2120 (13)	0.8370 (4)	0.088 (6)*	0.308 (6)
C11B	0.4239 (11)	0.2405 (12)	0.8838 (3)	0.080 (4)*	0.308 (6)
H11B	0.4289	0.3346	0.8943	0.096*	0.308 (6)
O1B	0.3470 (9)	0.116 (2)	0.6980 (4)	0.148 (9)*	0.308 (6)
O2B	0.1958 (7)	0.1460 (11)	0.6993 (3)	0.103 (3)*	0.308 (6)
C12B	0.2563 (6)	0.1302 (17)	0.6760 (3)	0.068 (3)*	0.308 (6)
C13B	0.2474 (8)	0.111 (3)	0.6263 (3)	0.085 (5)*	0.308 (6)
C14B	0.3233 (8)	0.0828 (13)	0.6055 (3)	0.110 (5)*	0.308 (6)
H14B	0.3838	0.0751	0.6231	0.132*	0.308 (6)
C15B	0.3109 (8)	0.0656 (17)	0.5587 (4)	0.144 (6)*	0.308 (6)
H15B	0.3636	0.0436	0.5444	0.173*	0.308 (6)
C16B	0.2255 (8)	0.0793 (15)	0.5321 (3)	0.122 (5)*	0.308 (6)
C17B	0.1482 (10)	0.092 (4)	0.5540 (5)	0.131 (9)*	0.308 (6)
H17B	0.0871	0.0862	0.5369	0.157*	0.308 (6)
C18B	0.1600 (7)	0.1134 (19)	0.6002 (4)	0.084 (4)*	0.308 (6)
H18B	0.1068	0.1302	0.6147	0.101*	0.308 (6)
C19B	0.2193 (12)	0.0783 (14)	0.4804 (4)	0.176 (6)*	0.308 (6)
H19C	0.1846	0.1624	0.4678	0.211*	0.308 (6)
H19D	0.2832	0.0841	0.4726	0.211*	0.308 (6)
C20B	0.1719 (10)	−0.0493 (14)	0.4583 (4)	0.150 (4)*	0.308 (6)
H20C	0.1042	−0.0435	0.4599	0.180*	0.308 (6)
H20D	0.1969	−0.1329	0.4758	0.180*	0.308 (6)
C21B	0.1845 (9)	−0.068 (2)	0.4092 (4)	0.197 (7)*	0.308 (6)
H21C	0.2172	0.0170	0.4005	0.237*	0.308 (6)
H21D	0.2280	−0.1470	0.4083	0.237*	0.308 (6)
C22B	0.1034 (8)	−0.0921 (19)	0.3719 (4)	0.169 (5)*	0.308 (6)
H22C	0.0646	−0.0066	0.3665	0.202*	0.308 (6)
H22D	0.0638	−0.1692	0.3804	0.202*	0.308 (6)
C23B	0.1438 (8)	−0.130 (2)	0.3296 (4)	0.169 (5)*	0.308 (6)
H23D	0.1818	−0.0516	0.3204	0.202*	0.308 (6)
H23E	0.1836	−0.2140	0.3349	0.202*	0.308 (6)
C24B	0.0605 (9)	−0.157 (2)	0.2937 (4)	0.180 (6)*	0.308 (6)
H24D	0.0815	−0.1898	0.2656	0.270*	0.308 (6)
H24G	0.0247	−0.0701	0.2873	0.270*	0.308 (6)
H24E	0.0208	−0.2281	0.3048	0.270*	0.308 (6)

### Atomic displacement parameters (Å^2^)

**Table:**

	*U*^11^	*U*^22^	*U*^33^	*U*^12^	*U*^13^	*U*^23^
N1A	0.162 (5)	0.174 (5)	0.093 (3)	−0.048 (4)	0.003 (2)	−0.042 (2)
C1A	0.165 (6)	0.177 (8)	0.137 (5)	−0.058 (5)	0.011 (4)	−0.031 (4)
C2A	0.112 (6)	0.146 (7)	0.104 (4)	−0.031 (3)	0.011 (2)	−0.025 (3)
C3A	0.132 (7)	0.131 (6)	0.105 (4)	−0.023 (3)	0.000 (2)	−0.005 (2)
C4A	0.143 (6)	0.111 (4)	0.131 (5)	−0.009 (3)	0.004 (3)	−0.014 (2)
C5A	0.080 (4)	0.103 (6)	0.100 (6)	−0.0027 (15)	0.011 (2)	−0.0106 (19)
C6A	0.082 (2)	0.097 (3)	0.091 (3)	0.0071 (14)	0.0105 (14)	−0.0054 (15)
C7A	0.095 (5)	0.146 (8)	0.098 (5)	0.012 (3)	0.0154 (19)	−0.001 (2)
C8A	0.161 (7)	0.145 (6)	0.155 (6)	−0.005 (3)	0.018 (4)	0.038 (4)
C9A	0.173 (7)	0.106 (4)	0.156 (7)	−0.017 (3)	0.015 (4)	0.008 (3)
C10A	0.097 (6)	0.106 (5)	0.125 (5)	−0.015 (2)	0.019 (3)	−0.011 (2)
C11A	0.140 (5)	0.119 (4)	0.149 (6)	−0.038 (3)	0.016 (3)	−0.036 (3)
O1A	0.0883 (19)	0.176 (5)	0.084 (2)	0.0302 (17)	0.0133 (10)	0.0084 (14)
O2A	0.110 (2)	0.135 (3)	0.108 (2)	0.0322 (19)	0.0273 (16)	0.014 (2)
C12A	0.113 (4)	0.118 (4)	0.140 (6)	0.019 (2)	0.034 (3)	0.027 (3)
C13A	0.104 (3)	0.091 (5)	0.103 (4)	0.017 (2)	0.0192 (19)	0.018 (2)
C14A	0.111 (3)	0.125 (4)	0.099 (3)	0.027 (3)	0.0207 (18)	0.013 (2)
C15A	0.127 (3)	0.153 (5)	0.100 (3)	0.041 (3)	0.022 (2)	0.008 (2)
C16A	0.142 (4)	0.122 (4)	0.100 (3)	0.028 (3)	0.015 (2)	0.003 (2)
C17A	0.126 (5)	0.152 (7)	0.114 (4)	0.033 (3)	−0.018 (3)	0.002 (3)
C18A	0.125 (4)	0.144 (5)	0.140 (6)	0.034 (3)	0.018 (3)	0.000 (4)
C19A	0.177 (5)	0.216 (8)	0.113 (4)	0.050 (5)	−0.004 (3)	−0.025 (4)
C20A	0.286 (9)	0.192 (7)	0.111 (4)	0.000 (6)	0.015 (4)	0.012 (4)
C21A	0.174 (5)	0.251 (9)	0.115 (4)	0.001 (6)	0.011 (3)	0.006 (4)
C22A	0.297 (9)	0.307 (10)	0.127 (4)	0.007 (7)	−0.028 (4)	0.004 (4)
C23A	0.297 (9)	0.307 (10)	0.127 (4)	0.007 (7)	−0.028 (4)	0.004 (4)
C24A	0.258 (9)	0.285 (11)	0.156 (6)	0.028 (8)	0.048 (6)	−0.035 (6)

### Geometric parameters (Å, º)

**Table:**

N1A—C1A	1.146 (6)	N1B—C1B	1.148 (9)
C1A—C2A	1.449 (5)	C1B—C2B	1.446 (7)
C2A—C11A	1.358 (6)	C2B—C11B	1.364 (7)
C2A—C3A	1.408 (6)	C2B—C3B	1.411 (8)
C3A—C4A	1.356 (5)	C3B—C4B	1.356 (7)
C3A—H3AA	0.9500	C3B—H3BA	0.9500
C4A—C5A	1.411 (6)	C4B—C5B	1.415 (8)
C4A—H4AA	0.9500	C4B—H4BA	0.9500
C5A—C6A	1.406 (5)	C5B—C6B	1.405 (8)
C5A—C10A	1.413 (5)	C5B—C10B	1.412 (7)
C6A—C7A	1.343 (5)	C6B—C7B	1.342 (8)
C6A—H6AA	0.9500	C6B—H6BA	0.9500
C7A—O1A	1.389 (5)	C7B—O1B	1.388 (8)
C7A—C8A	1.406 (6)	C7B—C8B	1.407 (8)
C8A—C9A	1.358 (6)	C8B—C9B	1.361 (8)
C8A—H8AA	0.9500	C8B—H8BA	0.9500
C9A—C10A	1.414 (6)	C9B—C10B	1.417 (8)
C9A—H9AA	0.9500	C9B—H9BA	0.9500
C10A—C11A	1.397 (6)	C10B—C11B	1.399 (8)
C11A—H11A	0.9500	C11B—H11B	0.9500
O1A—C12A	1.375 (5)	O1B—C12B	1.377 (8)
O2A—C12A	1.204 (5)	O2B—C12B	1.204 (7)
C12A—C13A	1.473 (6)	C12B—C13B	1.465 (8)
C13A—C14A	1.365 (5)	C13B—C14B	1.366 (8)
C13A—C18A	1.374 (5)	C13B—C18B	1.375 (7)
C14A—C15A	1.375 (5)	C14B—C15B	1.377 (8)
C14A—H14A	0.9500	C14B—H14B	0.9500
C15A—C16A	1.367 (5)	C15B—C16B	1.365 (9)
C15A—H15A	0.9500	C15B—H15B	0.9500
C16A—C17A	1.380 (7)	C16B—C17B	1.382 (10)
C16A—C19A	1.510 (6)	C16B—C19B	1.513 (9)
C17A—C18A	1.363 (7)	C17B—C18B	1.366 (9)
C17A—H17A	0.9500	C17B—H17B	0.9500
C18A—H18A	0.9500	C18B—H18B	0.9500
C19A—C20A	1.438 (7)	C19B—C20B	1.4992 (11)
C19A—H19A	0.9900	C19B—H19C	0.9900
C19A—H19B	0.9900	C19B—H19D	0.9900
C20A—C21A	1.485 (6)	C20B—C21B	1.4998 (11)
C20A—H20A	0.9900	C20B—H20C	0.9900
C20A—H20B	0.9900	C20B—H20D	0.9900
C21A—C22A	1.476 (8)	C21B—C22B	1.4990 (11)
C21A—H21A	0.9900	C21B—H21C	0.9900
C21A—H21B	0.9900	C21B—H21D	0.9900
C22A—C23A	1.574 (7)	C22B—C23B	1.5011 (11)
C22A—H22A	0.9900	C22B—H22C	0.9900
C22A—H22B	0.9900	C22B—H22D	0.9900
C23A—C24A	1.4984 (11)	C23B—C24B	1.4995 (11)
C23A—H23A	0.9900	C23B—H23D	0.9900
C23A—H23B	0.9900	C23B—H23E	0.9900
C24A—H24A	0.9800	C24B—H24D	0.9800
C24A—H24B	0.9800	C24B—H24G	0.9800
C24A—H24C	0.9800	C24B—H24E	0.9800
			
N1A—C1A—C2A	174.0 (11)	C3B—C2B—C1B	118.7 (8)
C11A—C2A—C3A	120.1 (5)	C4B—C3B—C2B	119.4 (8)
C11A—C2A—C1A	119.2 (6)	C4B—C3B—H3BA	120.3
C3A—C2A—C1A	120.5 (6)	C2B—C3B—H3BA	120.3
C4A—C3A—C2A	119.1 (5)	C3B—C4B—C5B	120.3 (8)
C4A—C3A—H3AA	120.5	C3B—C4B—H4BA	119.9
C2A—C3A—H3AA	120.5	C5B—C4B—H4BA	119.9
C3A—C4A—C5A	122.0 (6)	C6B—C5B—C10B	119.3 (8)
C3A—C4A—H4AA	119.0	C6B—C5B—C4B	120.6 (10)
C5A—C4A—H4AA	119.0	C10B—C5B—C4B	119.5 (8)
C6A—C5A—C4A	122.4 (5)	C7B—C6B—C5B	120.2 (10)
C6A—C5A—C10A	119.1 (4)	C7B—C6B—H6BA	119.9
C4A—C5A—C10A	118.4 (5)	C5B—C6B—H6BA	119.9
C7A—C6A—C5A	120.1 (4)	C6B—C7B—O1B	119.5 (10)
C7A—C6A—H6AA	119.9	C6B—C7B—C8B	121.8 (9)
C5A—C6A—H6AA	119.9	O1B—C7B—C8B	118.8 (9)
C6A—C7A—O1A	120.0 (6)	C9B—C8B—C7B	119.1 (8)
C6A—C7A—C8A	122.2 (5)	C9B—C8B—H8BA	120.4
O1A—C7A—C8A	117.5 (5)	C7B—C8B—H8BA	120.4
C9A—C8A—C7A	118.7 (6)	C8B—C9B—C10B	120.7 (8)
C9A—C8A—H8AA	120.6	C8B—C9B—H9BA	119.6
C7A—C8A—H8AA	120.6	C10B—C9B—H9BA	119.6
C8A—C9A—C10A	121.2 (6)	C11B—C10B—C5B	119.1 (7)
C8A—C9A—H9AA	119.4	C11B—C10B—C9B	122.0 (9)
C10A—C9A—H9AA	119.4	C5B—C10B—C9B	118.6 (7)
C11A—C10A—C5A	118.4 (5)	C2B—C11B—C10B	119.9 (8)
C11A—C10A—C9A	122.7 (6)	C2B—C11B—H11B	120.1
C5A—C10A—C9A	118.6 (5)	C10B—C11B—H11B	120.1
C2A—C11A—C10A	121.9 (5)	C12B—O1B—C7B	118.1 (13)
C2A—C11A—H11A	119.0	O2B—C12B—O1B	117.8 (9)
C10A—C11A—H11A	119.0	O2B—C12B—C13B	129.0 (8)
C12A—O1A—C7A	118.6 (6)	O1B—C12B—C13B	113.0 (7)
O2A—C12A—O1A	120.4 (5)	C14B—C13B—C18B	119.0 (7)
O2A—C12A—C13A	126.7 (5)	C14B—C13B—C12B	121.6 (7)
O1A—C12A—C13A	112.9 (5)	C18B—C13B—C12B	119.3 (8)
C14A—C13A—C18A	118.7 (5)	C13B—C14B—C15B	119.2 (9)
C14A—C13A—C12A	122.8 (4)	C13B—C14B—H14B	120.4
C18A—C13A—C12A	118.4 (5)	C15B—C14B—H14B	120.4
C13A—C14A—C15A	119.8 (4)	C16B—C15B—C14B	122.1 (9)
C13A—C14A—H14A	120.1	C16B—C15B—H15B	118.9
C15A—C14A—H14A	120.1	C14B—C15B—H15B	118.9
C16A—C15A—C14A	122.3 (4)	C15B—C16B—C17B	117.7 (8)
C16A—C15A—H15A	118.8	C15B—C16B—C19B	119.1 (9)
C14A—C15A—H15A	118.8	C17B—C16B—C19B	123.1 (9)
C15A—C16A—C17A	117.1 (4)	C18B—C17B—C16B	119.8 (10)
C15A—C16A—C19A	119.8 (4)	C18B—C17B—H17B	120.1
C17A—C16A—C19A	123.1 (4)	C16B—C17B—H17B	120.1
C18A—C17A—C16A	121.1 (5)	C17B—C18B—C13B	121.3 (9)
C18A—C17A—H17A	119.4	C17B—C18B—H18B	119.4
C16A—C17A—H17A	119.4	C13B—C18B—H18B	119.4
C17A—C18A—C13A	120.9 (6)	C20B—C19B—C16B	113.4 (9)
C17A—C18A—H18A	119.5	C20B—C19B—H19C	108.9
C13A—C18A—H18A	119.5	C16B—C19B—H19C	108.9
C20A—C19A—C16A	117.4 (5)	C20B—C19B—H19D	108.9
C20A—C19A—H19A	107.9	C16B—C19B—H19D	108.9
C16A—C19A—H19A	107.9	H19C—C19B—H19D	107.7
C20A—C19A—H19B	107.9	C19B—C20B—C21B	113.6 (9)
C16A—C19A—H19B	107.9	C19B—C20B—H20C	108.8
H19A—C19A—H19B	107.2	C21B—C20B—H20C	108.8
C19A—C20A—C21A	118.0 (6)	C19B—C20B—H20D	108.8
C19A—C20A—H20A	107.8	C21B—C20B—H20D	108.8
C21A—C20A—H20A	107.8	H20C—C20B—H20D	107.7
C19A—C20A—H20B	107.8	C22B—C21B—C20B	122.1 (10)
C21A—C20A—H20B	107.8	C22B—C21B—H21C	106.8
H20A—C20A—H20B	107.2	C20B—C21B—H21C	106.8
C22A—C21A—C20A	118.6 (6)	C22B—C21B—H21D	106.8
C22A—C21A—H21A	107.7	C20B—C21B—H21D	106.8
C20A—C21A—H21A	107.7	H21C—C21B—H21D	106.7
C22A—C21A—H21B	107.7	C21B—C22B—C23B	106.7 (8)
C20A—C21A—H21B	107.7	C21B—C22B—H22C	110.4
H21A—C21A—H21B	107.1	C23B—C22B—H22C	110.4
C21A—C22A—C23A	101.6 (6)	C21B—C22B—H22D	110.4
C21A—C22A—H22A	111.4	C23B—C22B—H22D	110.4
C23A—C22A—H22A	111.4	H22C—C22B—H22D	108.6
C21A—C22A—H22B	111.4	C24B—C23B—C22B	104.8 (8)
C23A—C22A—H22B	111.4	C24B—C23B—H23D	110.8
H22A—C22A—H22B	109.3	C22B—C23B—H23D	110.8
C24A—C23A—C22A	101.0 (6)	C24B—C23B—H23E	110.8
C24A—C23A—H23A	111.6	C22B—C23B—H23E	110.8
C22A—C23A—H23A	111.6	H23D—C23B—H23E	108.9
C24A—C23A—H23B	111.6	C23B—C24B—H24D	109.5
C22A—C23A—H23B	111.6	C23B—C24B—H24G	109.5
H23A—C23A—H23B	109.4	H24D—C24B—H24G	109.5
N1B—C1B—C2B	172 (2)	C23B—C24B—H24E	109.5
C11B—C2B—C3B	121.5 (8)	H24D—C24B—H24E	109.5
C11B—C2B—C1B	119.5 (8)	H24G—C24B—H24E	109.5
			
C11A—C2A—C3A—C4A	−3 (2)	C11B—C2B—C3B—C4B	4 (4)
C1A—C2A—C3A—C4A	−178.5 (13)	C1B—C2B—C3B—C4B	178 (2)
C2A—C3A—C4A—C5A	3 (2)	C2B—C3B—C4B—C5B	−7 (4)
C3A—C4A—C5A—C6A	−177.4 (15)	C3B—C4B—C5B—C6B	177 (3)
C3A—C4A—C5A—C10A	−2 (3)	C3B—C4B—C5B—C10B	6 (6)
C4A—C5A—C6A—C7A	175.3 (16)	C10B—C5B—C6B—C7B	−4 (6)
C10A—C5A—C6A—C7A	0 (3)	C4B—C5B—C6B—C7B	−175 (3)
C5A—C6A—C7A—O1A	174.3 (13)	C5B—C6B—C7B—O1B	−177 (3)
C5A—C6A—C7A—C8A	1 (2)	C5B—C6B—C7B—C8B	2 (5)
C6A—C7A—C8A—C9A	−2 (2)	C6B—C7B—C8B—C9B	3 (4)
O1A—C7A—C8A—C9A	−175.7 (12)	O1B—C7B—C8B—C9B	−178.5 (19)
C7A—C8A—C9A—C10A	3 (2)	C7B—C8B—C9B—C10B	−5 (4)
C6A—C5A—C10A—C11A	175.4 (16)	C6B—C5B—C10B—C11B	−173 (4)
C4A—C5A—C10A—C11A	0 (3)	C4B—C5B—C10B—C11B	−2 (7)
C6A—C5A—C10A—C9A	1 (3)	C6B—C5B—C10B—C9B	1 (7)
C4A—C5A—C10A—C9A	−174.6 (16)	C4B—C5B—C10B—C9B	172 (3)
C8A—C9A—C10A—C11A	−176.5 (16)	C8B—C9B—C10B—C11B	178 (3)
C8A—C9A—C10A—C5A	−2 (3)	C8B—C9B—C10B—C5B	4 (5)
C3A—C2A—C11A—C10A	1 (3)	C3B—C2B—C11B—C10B	−1 (4)
C1A—C2A—C11A—C10A	176.5 (15)	C1B—C2B—C11B—C10B	−174 (3)
C5A—C10A—C11A—C2A	1 (3)	C5B—C10B—C11B—C2B	0 (5)
C9A—C10A—C11A—C2A	174.8 (16)	C9B—C10B—C11B—C2B	−174 (3)
C6A—C7A—O1A—C12A	110.0 (13)	C6B—C7B—O1B—C12B	103 (3)
C8A—C7A—O1A—C12A	−76.0 (16)	C8B—C7B—O1B—C12B	−75 (3)
C7A—O1A—C12A—O2A	3.8 (16)	C7B—O1B—C12B—O2B	−12 (2)
C7A—O1A—C12A—C13A	−177.7 (9)	C7B—O1B—C12B—C13B	173.2 (17)
O2A—C12A—C13A—C14A	−178.4 (11)	O2B—C12B—C13B—C14B	−174.5 (18)
O1A—C12A—C13A—C14A	3.2 (17)	O1B—C12B—C13B—C14B	0 (3)
O2A—C12A—C13A—C18A	4 (2)	O2B—C12B—C13B—C18B	2 (4)
O1A—C12A—C13A—C18A	−174.6 (11)	O1B—C12B—C13B—C18B	176 (2)
C18A—C13A—C14A—C15A	−1.2 (15)	C18B—C13B—C14B—C15B	4 (3)
C12A—C13A—C14A—C15A	−178.9 (10)	C12B—C13B—C14B—C15B	180.0 (18)
C13A—C14A—C15A—C16A	−0.1 (12)	C13B—C14B—C15B—C16B	2 (2)
C14A—C15A—C16A—C17A	1.6 (13)	C14B—C15B—C16B—C17B	−9 (3)
C14A—C15A—C16A—C19A	−179.1 (7)	C14B—C15B—C16B—C19B	171.8 (12)
C15A—C16A—C17A—C18A	−2 (2)	C15B—C16B—C17B—C18B	10 (4)
C19A—C16A—C17A—C18A	178.8 (12)	C19B—C16B—C17B—C18B	−170 (2)
C16A—C17A—C18A—C13A	1 (2)	C16B—C17B—C18B—C13B	−5 (5)
C14A—C13A—C18A—C17A	1 (2)	C14B—C13B—C18B—C17B	−2 (4)
C12A—C13A—C18A—C17A	178.7 (14)	C12B—C13B—C18B—C17B	−178 (3)
C15A—C16A—C19A—C20A	−148.2 (8)	C15B—C16B—C19B—C20B	111.4 (16)
C17A—C16A—C19A—C20A	31.1 (15)	C17B—C16B—C19B—C20B	−68 (3)
C16A—C19A—C20A—C21A	173.8 (6)	C16B—C19B—C20B—C21B	−166.8 (12)
C19A—C20A—C21A—C22A	54.9 (12)	C19B—C20B—C21B—C22B	−129.0 (19)
C20A—C21A—C22A—C23A	156.5 (8)	C20B—C21B—C22B—C23B	−170.5 (17)
C21A—C22A—C23A—C24A	149.9 (9)	C21B—C22B—C23B—C24B	178.6 (14)

### Hydrogen-bond geometry (Å, º)

Cg1 and Cg2 are the centroids of the C2A–C5A/C10A/C11A and C13B–C18B rings, respectively.

**Table:**

*D*—H···*A*	*D*—H	H···*A*	*D*···*A*	*D*—H···*A*
C4*A*—H4*AA*···O2*A*^i^	0.95	2.44	3.303 (11)	149
C9*B*—H9*BA*···O2*B*^ii^	0.95	2.59	3.352 (11)	138
C14*B*—H14*B*···*Cg*1^iii^	0.95	2.85	3.708 (14)	151
C20*A*—H20*A*···*Cg*2^iv^	0.99	2.91	3.819 (14)	152
C19*B*—H19*C*···*Cg*2^iv^	0.99	2.88	3.746 (19)	146

Symmetry codes: (i) −*x*+1/2, *y*−1/2, −*z*+3/2; (ii) −*x*+1/2, *y*+1/2, −*z*+3/2; (iii) −*x*+1, *y*, −*z*+3/2; (iv) −*x*+1/2, −*y*+1/2, −*z*+1.
